# The oral microbiome and atherosclerosis: current evidence on association, mechanisms, and clinical implications

**DOI:** 10.3389/fimmu.2025.1640904

**Published:** 2025-10-15

**Authors:** Yan Xiao, Bin Gong, Jing Li, Nan Xu

**Affiliations:** ^1^ Oral Medicine Center, The First People’s Hospital of Yunnan Province, Kunming, Yunnan, China; ^2^ The Affiliated Hospital of Kunming University of Science and Technology, Kunming, Yunnan, China

**Keywords:** oral microbiome, atherosclerosis, periodontal disease, systemic inflammation, mechanisms, oral-gut axis, artificial intelligence, clinical guidelines

## Abstract

Atherosclerosis, the primary pathology of cardiovascular disease (CVD), is not fully explained by traditional risk factors. The oral microbiome has emerged as a key environmental contributor, yet the evidence for its role remains complex. This review moves beyond summarizing established associations to critically appraise the evidence, synthesize novel mechanistic insights, and outline future clinical frameworks. While traditional mechanisms such as direct bacteremia and systemic inflammation are covered, this review highlights emerging pathways including the oral-gut-vascular axis, epigenetic reprogramming ("inflammatory memory"), and the role of the multi-kingdom microbiome. We critically evaluate the evidence landscape, reconciling conflicting findings from observational studies, Mendelian randomization, and randomized controlled trials (RCTs) by systematically examining methodological heterogeneity. Furthermore, future directions are explored, focusing on the application of artificial intelligence (AI) for biomarker discovery and the development of novel interventions like engineered microbial therapeutics. Finally, the review translates scientific consensus into actionable clinical frameworks for interdisciplinary patient care. This comprehensive synthesis underscores the need to move towards mechanism-based, personalized strategies for CVD prevention.

## 1 Introduction

Atherosclerosis, a chronic inflammatory disease characterized by the buildup of lipid-rich plaques within arterial walls, stands as the primary underlying pathology for a spectrum of debilitating and often fatal cardiovascular diseases (CVDs), including coronary artery disease (CAD), myocardial infarction (MI), stroke, and peripheral artery disease (PAD) (1). Globally, CVDs represent the leading cause of morbidity and mortality, imposing an immense burden on public health systems and economies (2). While significant progress has been made in managing traditional CVD risk factors such as dyslipidemia (e.g., controlling LDL cholesterol), hypertension, cigarette smoking, and diabetes mellitus, these factors account for only approximately 50% to 70% of atherosclerotic events (3). This "residual risk" highlights the critical need to identify and understand additional contributing factors to atherogenesis.

In recent decades, the human microbiome—the collective communities of microorganisms inhabiting various body sites—has emerged as a significant environmental factor influencing host physiology, metabolism, and immunity (4). Extensive research has established compelling links between dysbiosis (imbalance) of the gut microbiome and the development and progression of CVD and its risk factors (5). Metabolites derived from gut microbial activity, such as trimethylamine N-oxide (TMAO), have been identified as potential mediators linking gut dysbiosis to increased cardiovascular risk (6).

While the gut microbiome has received considerable attention, the oral cavity harbors the second most diverse and complex microbial ecosystem in the human body, comprising bacteria, fungi, archaea, and viruses (7). The oral microbiome plays crucial roles in maintaining local homeostasis but is also increasingly implicated in systemic health and disease (8). A growing body of evidence, particularly focusing on periodontal diseases (PD)—chronic inflammatory conditions affecting the tooth-supporting structures initiated by bacterial biofilms—suggests that alterations in the oral microbiome may represent an independent risk factor for the development and progression of atherosclerosis (9).

However, despite a wealth of associative data, the field has reached an inflection point where the narrative must evolve from correlation to causation and functional understanding. The translation of this association into clinical practice is currently hindered by several critical challenges. The leap from association to causality remains tenuous, as higher-tier causal inference methods like Mendelian randomization have yielded conflicting results for major cardiovascular events. This uncertainty is mirrored in clinical trials; while periodontal therapy consistently improves surrogate inflammatory markers, a significant evidence gap persists, with major systematic reviews finding no conclusive proof that it prevents hard clinical endpoints like myocardial infarction or stroke (10). These discrepancies suggest that the underlying mechanisms are more intricate than previously assumed, likely involving multi-system pathways like the oral-gut-vascular axis and host epigenetic reprogramming. Progress is further complicated by significant methodological heterogeneity across studies and the translational challenges of preclinical models, which impede the synthesis of evidence and the discovery of reliable biomarkers.

This review, therefore, moves beyond a conventional summary of associations. It aims to critically appraise the evidence, integrate novel mechanistic insights from frontier research, and outline a more comprehensive framework that addresses these unresolved questions, ultimately charting a path toward improved clinical translation and practice.

## 2 The oral microbiome and atherosclerosis: epidemiological links

A substantial body of epidemiological research, spanning several decades and employing diverse study designs, points towards a consistent association between markers of poor oral health, particularly periodontal disease, and an increased risk of atherosclerosis and subsequent cardiovascular events.

### 2.1 Periodontal disease and atherosclerosis/CVD association

Periodontal diseases encompass a spectrum from gingivitis (reversible gum inflammation) to periodontitis (progressive destruction of tooth-supporting tissues, including bone) (11). Severe periodontitis affects a significant portion of the global adult population, estimated at 10-15% or even higher in certain demographics, making it one of the most common chronic inflammatory conditions worldwide (12).

Numerous observational studies, including cross-sectional, case-control, and prospective cohort designs, have reported a positive association between the presence and severity of PD and various manifestations of atherosclerotic CVD (9). Early studies suggested a 25% increased risk of atherosclerotic plaque formation in individuals with periodontitis (13). Meta-analyses and large cohort studies have reported hazard ratios (HR) or odds ratios (OR) typically ranging from 1.1 to 2.8 for incident CAD, stroke, MI, or composite CVD endpoints in individuals with PD compared to those without, even after adjusting for traditional cardiovascular risk factors (14). For instance, the PAROKRANK case-control study found an adjusted OR of 1.28 for a first MI in patients with PD (15), and a meta-analysis reported an overall relative risk (RR) of 1.18 for incident atherosclerotic CVD in populations with PD (10).

Recent, large-scale evidence has further solidified this association while highlighting its complexity. A comprehensive umbrella review in 2024, analyzing 41 systematic reviews, confirmed a consistent link between PD and CVD, with odds ratios ranging from 1.22 to 4.42 and risk ratios from 1.14 to 2.88. This review also identified a clear severity gradient, with the risk for CVD increasing from mild PD (RR = 1.09) to severe PD (RR = 1.25) (16). Furthermore, a massive prospective study using the UK Biobank cohort of nearly half a million participants found that self-reported periodontal disease was associated with a 9% increased risk of incident coronary artery disease. This study uniquely integrated polygenic risk scores and cardiac magnetic resonance imaging, demonstrating that the association is underpinned by both shared genetic predispositions and observable changes in cardiac structure (17).

The persistence of this association, albeit often attenuated, after statistical adjustment for shared confounders like smoking, diabetes, age, and socioeconomic status, lends support to the hypothesis that PD may contribute independently to cardiovascular risk (18). However, the strength and even the existence of this association can vary significantly across studies, a point which necessitates a critical appraisal of the underlying methodological landscape.

### 2.2 Oral microbiome composition and atherosclerosis markers

Moving beyond clinical diagnoses of PD, research has begun to directly investigate the relationship between the composition of the oral microbial community and subclinical or clinical markers of atherosclerosis. A study using data from the Northern Finland Birth Cohort of 1966 examined the link between the oral microbiome (assessed by 16S rRNA gene sequencing) and carotid intima-media thickness (cIMT), an established marker of atherosclerosis progression (1). This study found significant associations between oral microbiome diversity metrics (Shannon index, β-diversity) and cIMT specifically in middle-aged males, even after excluding individuals with major CVD risk factors. Furthermore, in this male sub-cohort, specific bacterial genera showed correlations with cIMT: *Prevotella*, *Megasphaera*, and *Veillonella* were positively associated, while *Absconditabacteria*, *Capnocytophaga*, *Gemella*, *Fusobacterium*, *Neisseria*, *Aggregatibacter*, *Tannerella*, *Treponema*, *Cardiobacterium*, and *Bacteroidales* showed inverse associations.

Other studies have identified specific taxa linked to clinical atherosclerosis. For example, a higher relative abundance of *Anaeroglobus* in oral samples was associated with symptomatic atherosclerosis compared to controls (7). In a Tunisian cohort, the genus *Eikenella* emerged as a key discriminant marker for CAD status and was negatively correlated with the Syntax score (a measure of CAD complexity), suggesting a potential role in earlier stages of atherosclerosis (7). Another study focusing on carotid atherosclerosis (CAS) found increased abundance of *Streptococcus*, *Lactobacillus*, and *Cutibacterium* in the saliva of affected individuals (6).

However, it is important to note that not all studies find dramatic shifts in the overall oral microbial structure between individuals with and without atherosclerosis. Some research indicates that the overall microbial community composition may be similar, suggesting that more subtle changes in specific taxa, their relative abundances, or their functional activities might be more relevant than large-scale ecosystem alterations (7). This lack of consensus on a specific "pro-atherogenic" microbial signature is a central challenge in the field and is largely driven by the methodological inconsistencies detailed below (**Table 1**).

**Table 1:** Key oral bacteria implicated in atherosclerosis and proposed mechanisms.

Bacterium/genus	Key proposed mechanisms	Key references
*Porphyromonas gingivalis (Pg)*	Translocation; Plaque presence (DNA, live); Endothelial invasion & dysfunction; Systemic inflammation (LPS, cytokines); Immune activation (macrophages, Th17); Molecular mimicry (HSPs); Foam cell formation; LDL/HDL oxidation; MMP induction (plaque destabilization); VSMC effects (proliferation, calcification); Platelet aggregation; Gut dysbiosis induction	(19)
*Aggregatibacter actinomycetemcomitans (Aa)*	Translocation; Plaque presence (DNA, live); Endothelial invasion & dysfunction; Systemic inflammation (LPS, LtxA, cytokines); Monocyte adhesion; Th17 activation; Foam cell formation	(9)
*Fusobacterium nucleatum (Fn)*	Translocation; Plaque presence (DNA); Endothelial adhesion/invasion (facilitated by Pg); Systemic inflammation (LPS, cytokines); Immune activation (macrophage M1 polarization); Gut dysbiosis induction; Plaque instability promotion (animal models); Altered lipid metabolism (hepatic); Foam cell formation	(20)
*Streptococcus* spp. (e.g., *S. mutans, S. sanguinis, S. gordonii, S. mitis*)	Translocation; Plaque presence (DNA); Endothelial invasion (*S. mutans*); Platelet adhesion & aggregation (Viridans group); Molecular mimicry (HSPs); Association with CAS; SCFA production	(21)
*Tannerella forsythia*	Translocation; Plaque presence (DNA); Systemic inflammation (LPS); Molecular mimicry (HSPs); Association with stroke risk	(22)
*Prevotella* spp. (*P. intermedia, P. nigrescens*)	Translocation; Plaque presence (DNA); Endothelial invasion (*P. intermedia*); Systemic inflammation (LPS); Molecular mimicry (HSPs); TMA production; SCFA production; Association with cIMT (positive); Association with high CVD risk (ML predictor)	(1)
*Veillonella* spp.	Plaque presence (DNA); Correlation between oral and plaque abundance; Association with cIMT (positive)	(23)
*Eikenella* spp. (*E. corrodens*)	Association with CAD presence/severity; Potential plaque presence (HACEK group); Pro-inflammatory effects (LPS on coronary endothelial cells)	(7)
*Anaeroglobus* spp.	Association with symptomatic atherosclerosis; Correlation with hs-CRP	(6)
*Treponema denticola*	Translocation; Plaque presence (DNA); Systemic inflammation (LPS); Molecular mimicry (HSPs); Association with cIMT (inverse)	(1)
*Campylobacter rectus*	Plaque presence (DNA)	(4)
*Chryseomonas/Pseudomonas luteola*	High abundance in plaque samples (discriminant genus)	(24)
*Lactobacillus* spp.	Association with CAS; SCFA production; Association with high CVD risk (LEfSe)	(6)

This table summarizes key findings based on the provided snippets. The strength of evidence varies for different bacteria and mechanisms. Abbreviations: LPS, Lipopolysaccharide; MMPs, Matrix Metalloproteinases; cIMT, Carotid Intima-Media Thickness; CAS, Carotid Atherosclerosis; VSMC, Vascular Smooth Muscle Cell.

### 2.3 Heterogeneity and methodological appraisal of epidemiological evidence

A critical review of the literature reveals that the inconsistencies in findings are often not a reflection of true biological variability but rather a direct consequence of a lack of methodological standardization across the field (25). This "standardization crisis" is a primary reason why synthesizing evidence is challenging and why a single, reproducible microbial signature for atherosclerotic risk remains elusive (26). Key sources of heterogeneity include:

#### 2.3.1 Inconsistent case definitions

Studies frequently employ different clinical criteria to define both the exposure ("periodontitis") and the outcome ("atherosclerosis") (25). Periodontitis might be classified based on varying thresholds for probing depth or clinical attachment loss, while atherosclerosis could be defined as a clinical event (e.g., myocardial infarction), a diagnosis from medical records, or a subclinical marker (e.g., cIMT). Comparing these fundamentally different biological states can lead to divergent findings (26).

#### 2.3.2 Disparities in sequencing technology

The choice of technology for microbial profiling profoundly impacts results. Early studies predominantly used 16S rRNA gene sequencing, which typically offers only genus-level resolution and provides no direct information on the functional capabilities of the microbiome (9). In contrast, shotgun metagenomic sequencing provides species- or even strain-level resolution and, crucially, a direct readout of the community's functional genetic potential. This transition from a taxonomic to a functional paradigm is critical, as findings based on 16S data may miss key functional drivers of disease (4).

#### 2.3.3 Lack of standardization in sample collection and confounder control

Studies use various sample types (e.g., saliva, subgingival plaque, tongue coating), which harbor distinct microbial communities and are not directly comparable (27). Furthermore, many observational studies fail to adequately measure and control for the numerous shared risk factors between PD and CVD (e.g., diet, socioeconomic status, specific genetic variants), leading to a high risk of spurious associations and false-positive findings (9, 28).

The impact of these factors is powerfully illustrated by large population studies. For example, analysis of the NHANES database revealed that associations between oral microbiome alpha diversity and CVD mortality were evident in Mexican American and Non-Hispanic White participants but were absent in other Hispanic and Non-Hispanic Black participants (29). This highlights that population-specific variations, likely driven by a combination of genetics, lifestyle, and other environmental factors, must be accounted for and demonstrates why a "one-size-fits-all" approach to this research is likely to fail. To visually summarize these key sources of variability, a comparative framework is presented below (**Table 2**).

**Table 2:** Framework for reconciling conflicting findings in epidemiological studies.

Methodological domain	Source of heterogeneity	Impact on findings
Exposure Definition	Varying clinical criteria for "periodontitis" (e.g., probing depth vs. attachment loss vs. self-report).	Studies with stricter definitions may show stronger associations; self-report may underestimate risk.
Outcome Definition	Diverse endpoints: clinical events (MI, stroke) vs. subclinical markers (cIMT, plaque burden).	Associations are often stronger for surrogate markers than for hard clinical endpoints.
Microbial Profiling	16S rRNA sequencing (genus-level, no functional data) vs. Shotgun Metagenomics (species-level, functional potential).	16S studies may miss functional drivers; results are not directly comparable across technologies.
Study Population	Differences in age, ethnicity, socioeconomic status, and baseline CVD risk across cohorts.	Associations can be population-specific (e.g., present in one ethnic group but not another).
Confounder Control	Inadequate or inconsistent statistical adjustment for shared risk factors (smoking, diabetes, diet).	Insufficient adjustment can lead to overestimated and spurious associations.

MI, Myocardial Infarction; cIMT, Carotid Intima-Media Thickness; CVD, Cardiovascular Disease.

#### 2.3.4 Translational challenges: from preclinical models to human evidence

A significant hurdle in establishing causality is the translational gap between animal models and human disease. While preclinical models, particularly in mice, are invaluable for dissecting specific molecular pathways (e.g., the effect of *P. gingivalis* LPS on foam cell formation), they have inherent limitations (30). These include differences in oral microbiome composition, immune responses, and the artificially accelerated nature of atherosclerosis induction compared to the decades-long process in humans (31). Consequently, findings from animal studies, while mechanistically insightful, may not be directly applicable to human physiology and require cautious interpretation when informing clinical risk. Overcoming these bench-to-bedside barriers is a critical step for future research.

### 2.4 Related oral conditions and atherosclerosis

The connection between oral inflammation/infection and atherosclerosis extends beyond periodontitis to other common oral conditions.

(1) Chronic Apical Periodontitis (CAP): CAP refers to chronic inflammation and potential bone destruction occurring around the root apex of a tooth, typically resulting from microbial infection of the dental pulp space (32). Like periodontitis, CAP represents a persistent source of chronic inflammation and bacterial challenge (33). Epidemiological evidence suggests a link between CAP and increased cardiovascular risk. A systematic review and meta-analysis found that patients diagnosed with atherosclerosis were almost three times more likely to also have CAP (OR = 2.94) (34). Studies using computed tomography to quantify atherosclerotic burden (e.g., aortic calcium scoring) have found a positive correlation between the presence and number of untreated CAP lesions and the extent of aortic atherosclerosis (35). Interestingly, this correlation was not observed for teeth with CAP that had received endodontic (root canal) treatment, suggesting that resolving the infection might mitigate the associated risk (36). While the evidence base is less extensive than for periodontitis, and causality remains unproven (37), the findings support the hypothesis that chronic inflammatory processes originating in the oral cavity, including those at the tooth apex, could significantly impact cardiovascular health (34).

(2) Dental Caries: Dental caries, or tooth decay, is another highly prevalent oral disease initiated by bacterial activity, primarily the fermentation of dietary sugars by bacteria like *Streptococcus mutans*, leading to demineralization of tooth structures (38). If untreated, caries can progress to involve the dental pulp, causing infection and inflammation (33). Emerging evidence suggests a potential link between dental caries and CVD risk (39). The Atherosclerosis Risk in Communities (ARIC) study, following a large cohort, found that participants with one or more dental caries at baseline had a significantly increased risk of incident ischemic stroke (adjusted HR 1.40) and all-cause mortality (adjusted HR 1.13) over follow-up, although no significant association was found for CHD events (40). A dose-response relationship was also observed, with increasing numbers of decayed, missing, or filled surfaces correlating with higher stroke and mortality risk (41). Mechanistically, it is proposed that bacteria from carious lesions, such as *S. mutans*, can enter the bloodstream and contribute to systemic inflammation or directly colonize atheromas, as *S. mutans* DNA has been detected in atherosclerotic plaques and heart valves (33). Furthermore, poor oral hygiene practices, which increase the risk of both caries and periodontitis, are independently associated with higher CVD risk (42).

## 3 New frontiers in mechanistic understanding

The scientific narrative connecting the oral microbiome to atherosclerosis is undergoing a profound transformation, moving beyond the traditional focus on a few key periodontal pathogens to explore systems-level mechanisms (43). This section details four novel frontiers that are reshaping our understanding: integrative multi-omics, the epigenetic interface, the multi-kingdom ecology of the oral cavity, and the oral-gut-vascular axis as a unified pathway.

### 3.1 Integrative multi-omics approaches: from correlation to function

The evolution of microbiome research is marked by a critical paradigm shift from compositional analysis to functional investigation (44). Early studies, predominantly relying on 16S ribosomal RNA (rRNA) gene sequencing, were instrumental in identifying bacterial taxa associated with atherosclerosis but provided limited insight into the biological activities of the microbial community (45). The contemporary frontier lies in applying integrative multi-omics—combining genomics, transcriptomics, proteomics, and metabolomics—to create a holistic view of the host-microbiome interactome (46).

This functional approach is predicated on the understanding that the metabolic output of the microbiome and its intricate crosstalk with host pathways are more mechanistically relevant to disease pathogenesis than the mere presence of specific microbes (47). For example, a landmark multi-omics analysis of gut microbiota in atherosclerosis identified five distinct "microbe-metabolite-host gene" tripartite networks causally linked to the disease (48). Similarly, a study integrating salivary microbiome and metabolome analysis in patients with carotid atherosclerosis (CAS) identified distinctive microbial and metabolic signatures that correlated with increased carotid intima-media thickness (IMT) and serum hs-CRP (6). These studies exemplify the crucial shift from identifying "who is there" to understanding "what are they doing," a transition essential for developing functionally targeted therapies (49).

### 3.2 The epigenetic interface: how oral microbes reprogram host inflammatory pathways

Epigenetics offers a compelling molecular mechanism to explain how transient microbial exposures can lead to stable, long-term changes in host gene expression that drive chronic diseases like atherosclerosis (50). The oral pathobiont *Porphyromonas gingivalis* has emerged as a particularly sophisticated "epigenetic engineer" (51). Its lipopolysaccharide (LPS) has been shown to simultaneously decrease the expression of DNMT1 (removing repressive "brakes" on inflammatory genes) while upregulating the histone acetyltransferase (HAT) p300 and the master inflammatory transcription factor NF-κB (hitting the transcriptional "accelerator") (52).

This epigenetic reprogramming can have remarkably persistent effects, leading to a phenomenon of "inflammatory memory" or "trained immunity" (53). Direct evidence for this comes from a bone marrow transplant model, where healthy recipient mice receiving bone marrow from *P. gingivalis*-infected donors developed significantly greater atherosclerotic lesions, despite never being directly exposed to the pathogen (54). This demonstrates that the memory of a past oral infection can be encoded in the epigenome of hematopoietic stem cells, creating a long-lasting, pathogen-independent cardiovascular risk (55). This has profound clinical implications, suggesting that simply eliminating the active infection may be insufficient to fully reverse the associated cardiovascular risk (56).

### 3.3 The oral-gut-vascular axis: a unified mechanistic pathway

While direct bacteremia is a recognized mechanism, emerging evidence points to a more insidious and chronic pathway: the oral-gut-vascular axis (57). This axis describes a multi-stage process where oral pathobionts, ingested with saliva, first disrupt the distal gut ecosystem, leading to systemic pathology (58). In the context of severe periodontitis, an individual may swallow up to 10¹² oral bacteria daily, including key pathobionts like *P. gingivalis* that can survive gastric transit (59).

The arrival of this large, dysbiotic oral inoculum can induce gut dysbiosis and, critically, lead to a failure of the intestinal epithelial barrier, a condition often termed "leaky gut" (60). Mechanistic studies have shown this is caused by a decreased expression of key tight junction protein genes in intestinal tissues (61). This increased permeability allows microbial products, such as LPS, to translocate from the gut into the bloodstream, contributing to the state of chronic, low-grade "metabolic endotoxemia" that is a hallmark of atherosclerosis (62). Furthermore, this *P. gingivalis*-induced gut dysbiosis alters the metabolic output of the microbial community, most notably by enriching for bacteria that are proficient producers of trimethylamine (TMA), the precursor to the pro-atherogenic molecule TMAO (63).

### 3.4 Beyond bacteria: the emerging roles of the oral virome and mycobiome

The vast majority of research has been bacterio-centric, leaving a significant knowledge gap concerning the oral virome (viruses) and mycobiome (fungi) (64). Recognizing the oral microbiome as a multi-kingdom community is a critical new frontier, as the true drivers of dysbiosis may lie in the disruption of cross-kingdom interactions (65). The oral virome, dominated by bacteriophages, acts as a powerful regulator of the bacterial community (46). While direct evidence linking the oral virome to atherosclerosis is sparse, alterations in the *gut* virome have been associated with multiple cardiovascular conditions, establishing a compelling precedent for its investigation (65).

Similarly, research on the oral mycobiome is in its infancy. However, a pioneering study on the *gut* mycobiome found a significant negative correlation between the fungal genus *Mucor* and carotid intima-media thickness (cIMT), suggesting a potential protective role for certain fungi in vascular health (66). This finding is profound, as it establishes for the first time that non-bacterial microbes can be linked to a key marker of cardiovascular risk and underscores the necessity of a multi-kingdom approach in future research (67).

### 3.5 Convergence on core atherogenic pathways

The frontier mechanisms detailed above—from multi-system dysbiosis via the oral-gut axis to long-term epigenetic reprogramming of immune cells—ultimately converge and manifest through several well-established downstream atherogenic pathways within the vasculature. These core processes include:

#### 3.5.1 Direct vascular effects following bacteremia

**Figure 1:**
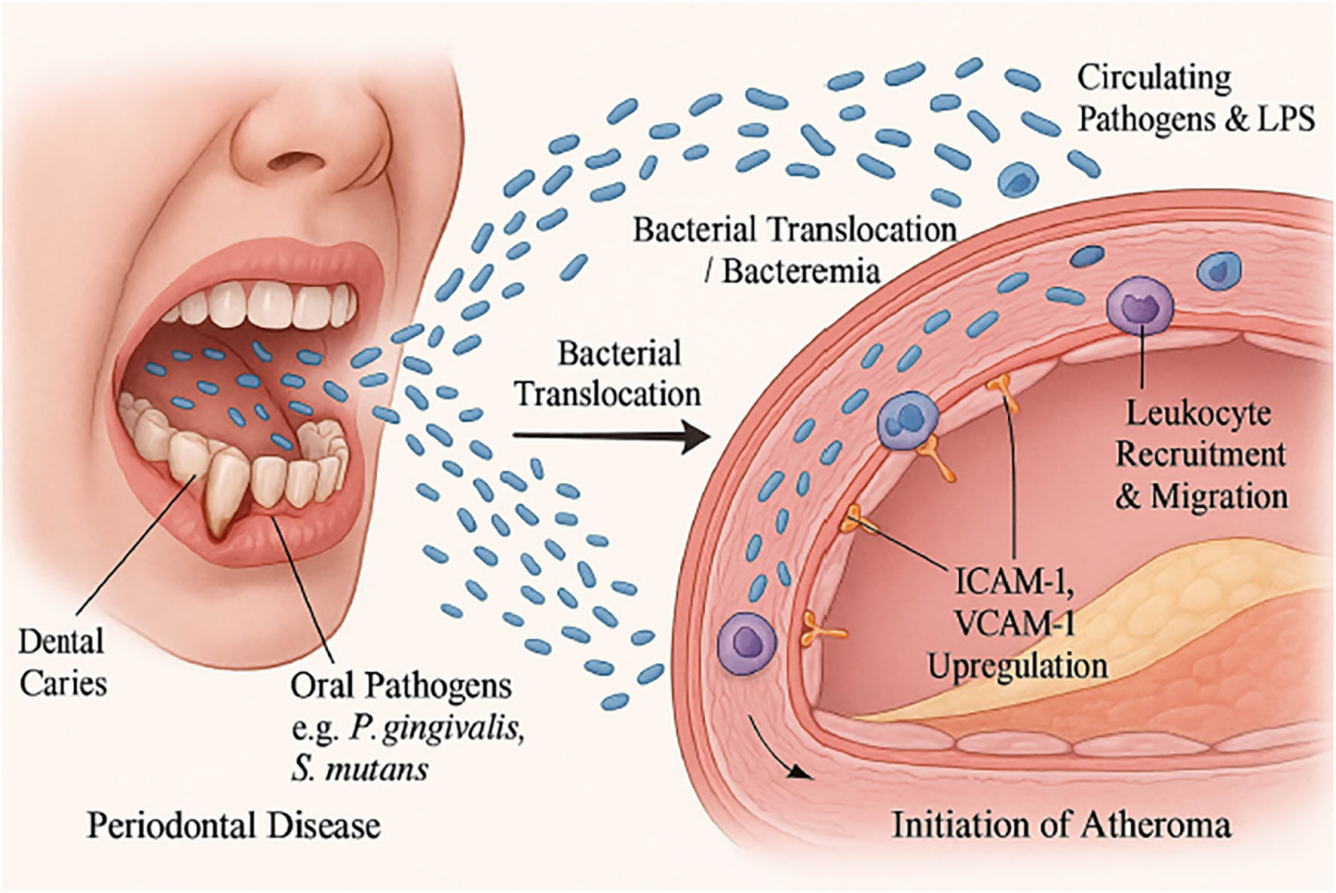
Bacterial translocation and direct endothelial activation in atherogenesis. Oral pathogens from dental caries and periodontal disease (e.g., *Porphyromonas gingivalis*, *Streptococcus mutans*) and their LPS can translocate into the bloodstream (bacteremia), triggering endothelial activation with ICAM-1/VCAM-1 upregulation, leukocyte recruitment and migration, and the initiation of atheroma.

A primary proposed mechanism involves the translocation of oral bacteria or their components into the systemic circulation, a phenomenon known as bacteremia (30). Once in the circulation, certain oral bacteria possess the ability to directly adhere to and invade vascular endothelial cells (34). This bacterial invasion can trigger endothelial dysfunction, a critical early event in atherogenesis, by upregulating adhesion molecules and pro-inflammatory cytokines(9, 37, 38) (**Figure 1**).

#### 3.5.2 Sustained systemic inflammation and immune dysregulation

**Figure 2:**
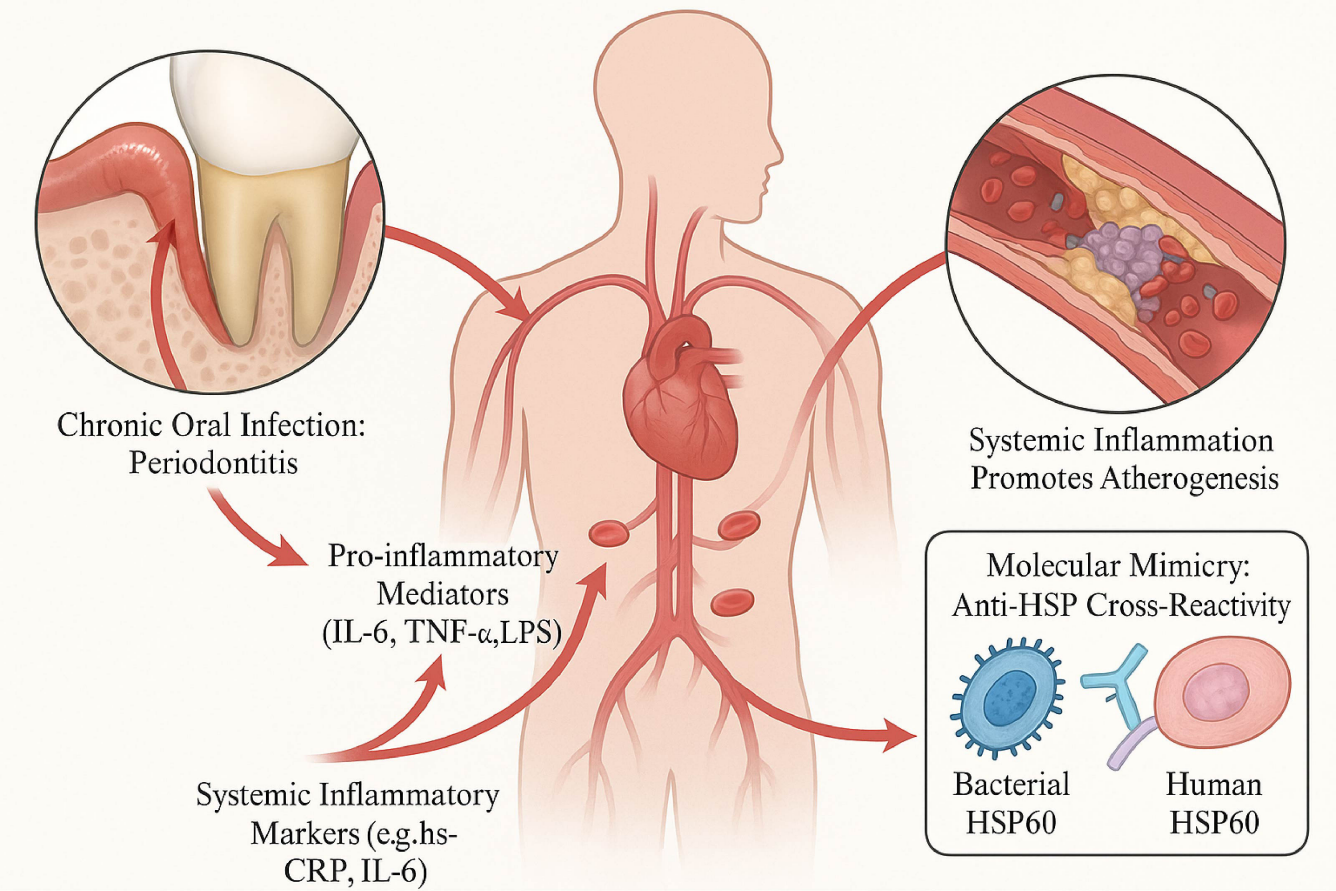
Systemic inflammation and immune dysregulation linking oral infections to atherosclerosis. This diagram outlines how chronic oral infections, particularly periodontitis, can promote atherosclerosis through systemic pathways. The infected oral tissues release pro-inflammatory mediators (e.g., IL-6, TNF-α, LPS) into the systemic circulation. This leads to an elevation of systemic inflammatory markers, such as hs-CRP and IL-6. This sustained systemic inflammation promotes atherogenesis in distant arterial sites. An additional mechanism depicted is molecular mimicry, where the immune response to bacterial Heat Shock Proteins (e.g., bacterial HSP60) can cross-react with human HSP60 expressed on stressed endothelial cells, further contributing to vascular damage and inflammation, thereby perpetuating a pro-atherogenic state throughout the vasculature.

Oral infections act as a chronic source of systemic inflammation, releasing pro-inflammatory mediators and bacterial components like LPS into the circulation (9, 39, 40). This chronic, low-grade systemic inflammation is reflected in elevated biomarkers like hs-CRP and drives atherogenesis at multiple stages(4, 41, 42). This process also involves adverse immune responses, such as molecular mimicry related to Heat Shock Proteins (HSPs), which can perpetuate endothelial damage(1, 44) (**Figure 2**).

#### 3.5.3 Destabilization of atherosclerotic plaques

Oral pathogens and their products, found within atheromas, directly contribute to plaque vulnerability. They promote the transformation of macrophages into foam cells, which form the plaque's necrotic core (35, 47). Concurrently, they induce the secretion of matrix metalloproteinases (MMPs) that degrade the protective fibrous cap, and they can alter vascular smooth muscle cell (VSMC) behavior, collectively increasing the risk of plaque rupture(35, 51, 54).

#### 3.5.4 Pro-thrombotic and metabolic dysregulation

**Figure 3:**
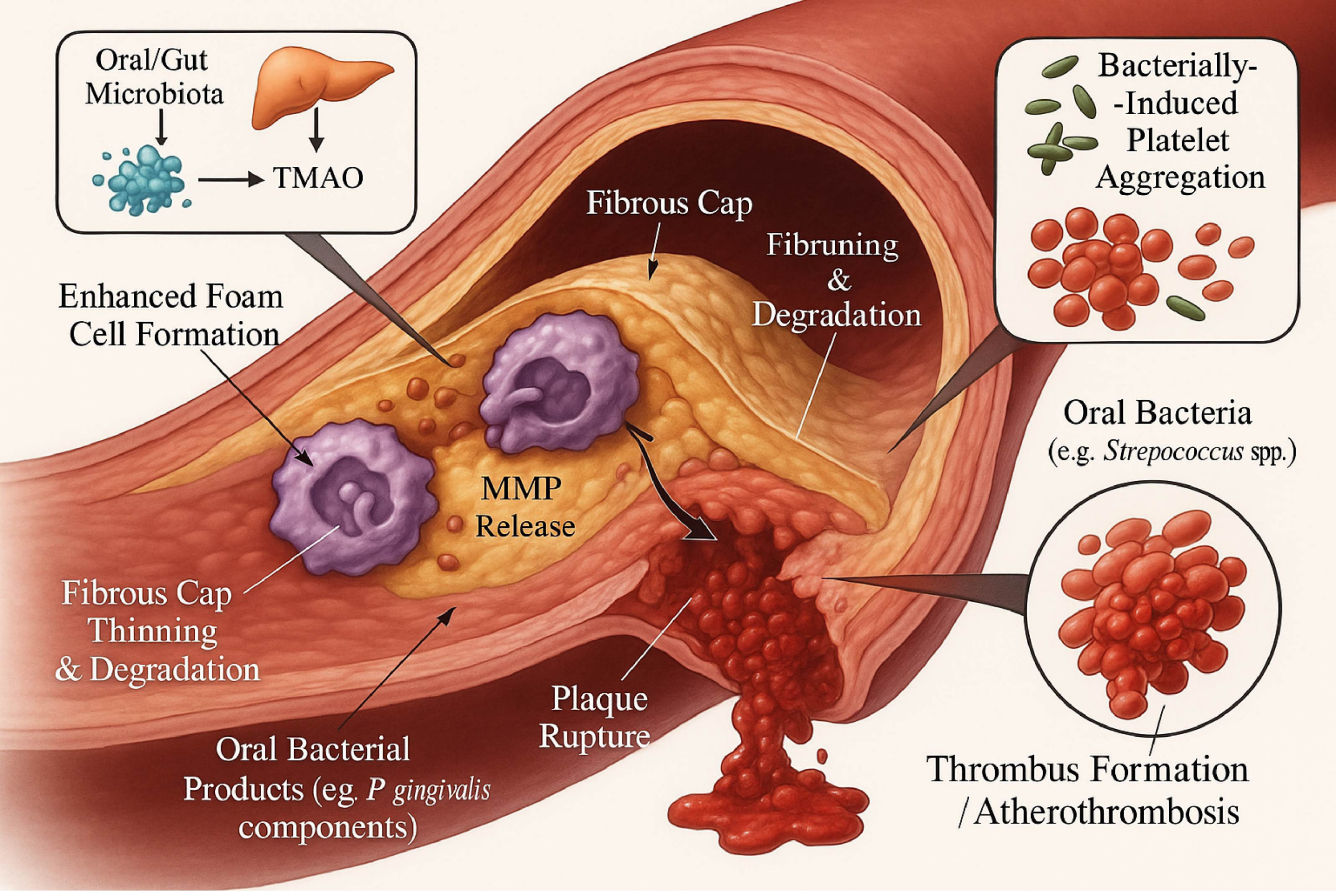
Impact of oral microbiota on atherosclerotic plaque progression, vulnerability, and thrombosis. This figure details several mechanisms by which the oral microbiome can influence established atherosclerotic plaques, leading to increased instability and risk of thrombosis. One pathway involves metabolites from the oral/gut microbiota, such as TMAO (produced from TMA by the liver), which enhances foam cell formation within the plaque. Oral bacterial products, like those from P. gingivalis, also contribute to enhanced foam cell formation and induce the release of Matrix Metalloproteinases (MMPs) by macrophages. These MMPs degrade the fibrous cap of the plaque, leading to its thinning and an increased risk of plaque rupture. Concurrently, certain oral bacteria (e.g., Streptococcus spp.) can directly induce platelet aggregation. The culmination of these processes—fibrous cap degradation and enhanced platelet activity—culminates in plaque rupture and subsequent thrombus formation (atherothrombosis), increasing the risk of acute cardiovascular events.

Beyond inflammation, oral microbes like *P. gingivalis* and viridans group streptococci can directly enhance platelet aggregation, increasing the risk of atherothrombosis upon plaque rupture (9, 35, 59). They also contribute to metabolic shifts, such as the oxidation of lipoproteins, which further fuels the atherogenic process (4, 33) (**Figure 3**).

## 4 Biomarker discovery and predictive modeling

The search for reliable oral biomarkers to predict cardiovascular risk has evolved alongside our mechanistic understanding. Early research focused on identifying specific bacterial taxa or changes in overall microbial diversity associated with atherosclerotic markers. While these studies provided foundational proof-of-concept, linking genera like.

*Anaeroglobus* to symptomatic atherosclerosis or altered diversity metrics to increased cIMT, their clinical predictive power has been limited. The sheer complexity of the microbiome, combined with the methodological heterogeneities discussed previously, necessitates more sophisticated analytical tools to translate these vast datasets into clinically actionable risk stratification.

Traditional CVD risk prediction models, such as the Framingham Risk Score, are based on a limited set of conventional risk factors and are ill-equipped to handle the high-dimensional, non-linear data generated by microbiome studies (68). This has created a critical need for more advanced analytical tools, a need that is being met by artificial intelligence (AI) and machine learning (ML) (69).

AI/ML represents a paradigm shift in risk prediction, offering scalable solutions that can integrate diverse data types to identify subtle patterns and build highly accurate models (69). This approach has been applied to the oral microbiome with remarkable success. For instance, a study using a Random Forest classifier trained on salivary microbiota data successfully identified a signature of 43 OTUs that could distinguish patients with atherosclerotic cardiovascular disease (ACVD) from controls with an outstanding Area Under the Curve (AUC) of 0.933. Other models integrating oral microbiome data with clinical parameters now report over 90% accuracy in CVD prediction, and the first AI-driven cardiovascular diagnostic algorithms received FDA approval in 2023, signaling a major clinical trend (70).

Despite their predictive power, a major barrier to the clinical adoption of many ML models is their "black box" nature; they can make accurate predictions, but the biological reasoning behind them is often opaque (71). A prediction is of limited clinical utility if a physician cannot understand *why* the model has classified a patient as high-risk. To address this, the field is rapidly moving towards eXplainable AI (XAI) (72). Techniques such as SHAP (Shapley Additive exPlanations) are being integrated into ML pipelines to provide insights into model predictions. These methods can assign a contribution value to each input feature (e.g., each bacterial taxon), effectively highlighting the specific microbes that are driving the model's decision (45). This not only increases clinical trust and transparency but also allows for the discovery of novel, biologically relevant biomarkers from the data itself.

The ultimate vision is the deep integration of diverse clinical data streams—microbiome profiles, proteomics, metabolomics, genomic data, and electronic health records—into unified predictive models (45). Realizing this vision requires breaking down the traditional silos between medical and dental care, creating systems where a patient's periodontal status and oral microbiome data can be seamlessly integrated with their lipid panel and blood pressure readings to generate a holistic, personalized, and highly accurate cardiovascular risk profile (45).

## 5 Periodontal intervention trials: a critical look at the evidence gap

To bridge the gap between observed associations and potential causality, randomized controlled trials (RCTs) investigating the effects of periodontal therapy on cardiovascular outcomes are essential (73). If treating periodontal disease leads to a reduction in CVD risk or events, it would provide strong support for a causal link and potentially introduce a novel preventive strategy (**Table 3**).

**Table 3:** Summary of periodontal intervention trials assessing cardiovascular markers/outcomes (based on systematic reviews/meta-analyses).

Outcome(s) assessed	Study/review type	Key references	Key findings/Effect size	Certainty of evidence	Limitations noted
Inflammatory Markers (hs-CRP, IL-6, TNF-α, Fibrinogen)	MA / SR	(74)	Significant reductions post-NSPT (e.g., hsCRP WMD ~ -0.5 to -1.2 mg/L; IL-6 WMD ~ -0.5 to -0.9 ng/L)	Moderate (for CRP, IL-6) (75)	Heterogeneity, short follow-up
Endothelial Function (FMD)	MA / SR / RCT	(76)	Significant improvement post-NSPT	Moderate (74)	Surrogate outcome, short follow-up
Lipid Profiles	MA / SR	(74)	Limited/inconsistent effects; possible small improvements in total cholesterol, HDL, triglycerides (esp. with comorbidities); reduced oxLDL/VLDL in one review	Low/Moderate (77)	Heterogeneity, inconsistent findings
Blood Pressure	MA / SR	(78)	Potential modest reduction	Limited (79)	Few studies, heterogeneity
Clinical Events (MACE, Mortality)	SR (Cochrane) / RCTs	(73)	Insufficient evidence to determine effect; No significant reduction demonstrated in existing trials (e.g., PAVE)	Very Low (80)	Few trials, small size, short follow-up, high risk of bias, focus on surrogates.

### 5.1 Consistent improvements in surrogate markers of cardiovascular risk

A considerable number of intervention studies have focused on the impact of periodontal treatment, primarily non-surgical periodontal therapy (NSPT), on intermediate or surrogate markers of cardiovascular risk. Systematic reviews and meta-analyses consistently demonstrate that NSPT leads to statistically significant reductions in key systemic inflammatory markers, such as high-sensitivity C-reactive protein (hs-CRP) and Interleukin-6 (IL-6) (81, 75). The effects of periodontal treatment on lipid profiles and blood pressure are less consistent, though some studies suggest potential benefits (74, 82).

Despite these consistent and encouraging improvements in surrogate markers, translating these biological effects into a demonstrable reduction in clinical cardiovascular events has proven to be a major, persistent challenge for the field.

### 5.2 The evidence gap: a lack of benefit in hard clinical endpoints

The critical question remains whether treating periodontal disease translates into a reduction in actual clinical cardiovascular events, such as MI, stroke, or cardiovascular death. Currently, the evidence from RCTs is insufficient to answer this question definitively (73).

Authoritative systematic reviews, including those by Cochrane, have consistently concluded that the available evidence is of very low certainty and is insufficient to support or refute the hypothesis that periodontal therapy prevents the occurrence or recurrence of CVD events (83, 84). The 2022 Cochrane review, for example, identified only two RCTs that met its strict inclusion criteria for assessing the prevention of cardiovascular events:

The López 2012 trial, which focused on primary prevention in patients with metabolic syndrome, provided insufficient evidence to draw any conclusions (85).

The PAVE 2008 trial, a secondary prevention study in patients with established CVD, also demonstrated no reliable evidence of benefit, a finding severely hampered by methodological issues, including a very high dropout rate (86, 87).

This profound disconnect between the consistent positive effects on surrogate markers and the lack of proven benefit on hard clinical endpoints represents a central challenge. This gap is likely not because the biological link is non-existent, but because existing trials have been plagued by significant design limitations, including:

Insufficient Power and Duration: Trials have generally been too small and too short (months to a few years) to detect statistically significant differences in relatively infrequent clinical events that develop over decades (73, 80).

Focus on Surrogate Outcomes: Many trials were primarily designed to assess effects on biomarkers rather than being powered for clinical events (88). While surrogate markers like CRP are associated with risk, their modification does not always translate to improved clinical outcomes.

Methodological Quality: Key studies have been assessed as having a high risk of bias, poor reporting quality, and a tendency to downplay non-significant primary outcomes in favor of secondary findings (70).

Potential for Unmeasured Confounding: Even in RCTs, factors like long-term lifestyle changes or adherence to medication may act as unmeasured confounders, creating potential for collider bias (89).

### 6 Addressing confounding and the elusive question of causality

### 6.1 The challenge of shared risk factors and confounding

A central challenge in interpreting the link between oral health and atherosclerosis is the issue of confounding (11). Periodontal disease and cardiovascular disease share numerous common risk factors, making it difficult to disentangle whether PD is an independent cause of CVD or merely a marker of a shared underlying susceptibility or exposure profile. Key shared risk factors include cigarette smoking (1), diabetes mellitus (90), age (91), socioeconomic status (SES) (92), and obesity/body mass index (BMI) (15).

Despite the challenge, many well-conducted observational studies have found that the association between PD and CVD persists even after statistical adjustment for these known shared risk factors (15). This residual association is often interpreted as supporting an independent, potentially causal, role for PD (18). However, the possibility of residual confounding from imprecisely measured or unmeasured factors remains a significant limitation of observational research (93, 94). To overcome these limitations and probe the question of causality more directly, researchers have turned to more advanced methods.

### 6.2 Advanced causal inference: conflicting evidence from mendelian randomization

Mendelian randomization (MR) is a powerful genetic epidemiological method that uses genetic variants as instrumental variables to assess the causal effect of an exposure on an outcome, thereby minimizing the influence of traditional confounding. In recent years, MR studies have been applied to the PD-CVD link, yielding critical but conflicting results.

On one hand, a landmark MR study provided the first genetic evidence for a causal relationship between periodontitis and hypertension (95). The study found that a genetic predisposition to periodontitis was causally associated with increased blood pressure, a major risk factor for atherosclerosis, a finding further supported by a small interventional trial showing that periodontal therapy reduced blood pressure (95).

On the other hand, when examining major atherosclerotic events directly, the evidence for causality disappears. More recent, large-scale MR studies have found no significant causal relationship between a genetic liability to periodontitis and the risk of coronary artery disease (CAD), myocardial infarction (MI), or stroke (96). For example, one major study reported an odds ratio (OR) for CAD of 1.01 (95% CI = 0.99-1.03) and for stroke of 0.99 (95% CI = 0.97-1.02), indicating a null effect (97). This discrepancy suggests that while oral bacteria and inflammation may causally influence certain risk factors like blood pressure, they may not be a direct causal driver of ultimate atherothrombotic events, or their effect is too small to be detected even with this powerful method.

### 6.3 Critical appraisal using the bradford hill criteria

Another framework for evaluating causality is the Bradford Hill criteria, which assesses factors like strength, consistency, specificity, and biological plausibility. When the PD-CVD link has been formally assessed against this framework, the evidence for causality has been found to be weak.

A systematic umbrella review applying these criteria to the existing body of meta-analyses concluded that the relationship failed to meet several key standards (74). Specifically, the criteria for 'consistency' and 'specificity' were not met due to the numerous inconsistencies across studies (as discussed in Section 2.3) and the fact that periodontitis is associated with multiple systemic conditions, not just atherosclerosis (74). While there is strong evidence for biological plausibility, the overall assessment concluded that the Bradford Hill criteria do not support a causal relationship based on current evidence.

In summary, while a biologically plausible association between periodontal disease and atherosclerotic CVD persists after adjusting for known confounders (12, 15), the current body of evidence does not support a direct causal relationship for major clinical events. The lack of definitive proof from RCTs, the conflicting results from Mendelian randomization, and the failure to satisfy the Bradford Hill criteria all point towards a relationship that is more complex than a simple cause-and-effect (12). This underscores the current scientific consensus: periodontitis should be considered a contributing factor and a marker of systemic inflammatory risk, but not yet a proven causal agent for atherosclerosis itself (12).

## 7 Clinical implications and future directions: from guidelines to actionable frameworks

The accumulating epidemiological and mechanistic evidence has propelled the oral-cardiovascular link from a research curiosity to a matter of clinical importance. In response, major professional societies in both cardiology and periodontology have collaborated to translate this scientific knowledge into practical, evidence-based guidelines for patient care, paving the way for integrated care models and novel therapeutic strategies.

### 7.1 Joint society guidelines for interdisciplinary patient management

There is now a clear international consensus, spearheaded by organizations like the European Federation of Periodontology (EFP) and the World Heart Federation (WHF), acknowledging the independent association between periodontitis and atherosclerotic cardiovascular disease (74). This has led to the development of high-level clinical practice guidelines, such as the EFP S3 Level Clinical Practice Guideline, which are based on formal evidence from multiple systematic reviews (98). These guidelines establish a new standard of care that relies on bidirectional screening, referral, and communication between medical and dental teams.

### 7.2 Key recommendations for the oral healthcare team (dentists, hygienists)

#### 7.2.1 Cardiovascular risk screening

Oral health professionals are encouraged to take an active role in primary CVD prevention by assessing major risk factors (e.g., smoking, diabetes, hypertension) and using validated risk assessment tools (99, 100).

#### 7.2.2 Patient education and referral

Patients diagnosed with periodontitis should be explicitly informed of their increased risk for future cardiovascular events. Those identified with uncontrolled CVD risk factors should be formally referred to their physician for co-management (74, 101).

#### 7.2.3 Safety of periodontal therapy

Periodontal treatment is deemed safe for the vast majority of patients with stable CVD. Any modification to antithrombotic therapy must be made only after consultation with the prescribing physician or cardiologist (74).

### 7.3 Actionable clinical framework for oral healthcare teams

#### 7.3.1 Standardized screening protocol

Implement a brief, chairside CVD risk questionnaire for all new adult patients, focusing on self-reported hypertension, diabetes, smoking status, and family history of CVD. Flag patients with two or more risk factors for further discussion (99, 102).

#### 7.3.2 Formal referral pathway

Utilize a pre-defined referral letter template for patients identified as high-risk. The letter should clearly state the diagnosis of periodontitis and request a cardiovascular assessment from their general physician or cardiologist (103).

#### 7.3.3 Patient education material

Provide patients with a standardized, one-page handout explaining the link between oral health and heart health in simple terms, reinforcing the importance of the medical referral (104).

### 7.4 Key recommendations for the medical team (cardiologists, general physicians)

#### 7.4.1 Oral health screening

Physicians managing patients with CVD should incorporate basic oral health screening into their routine evaluation, asking about signs of periodontitis like bleeding gums or tooth mobility (74).

#### 7.4.2 Patient education and referral

CVD patients should be informed that periodontitis can negatively impact their cardiovascular health. All patients with a new CVD diagnosis should be referred for a comprehensive periodontal examination (105).

#### 7.4.3 Interdisciplinary communication

Physicians should be prepared to liaise with the patient's dental team, particularly regarding the management of antithrombotic therapy prior to any planned invasive dental procedures (106).

### 7.5 Actionable clinical framework for medical teams

#### 7.5.1 Simplified oral health assessment tool

Incorporate a two-question screening into the routine patient intake (1): "Have your gums bled in the last month when brushing?" and (2) "Are any of your teeth loose?". A 'yes' to either question triggers a formal dental referral (107).

#### 7.5.2 Indications for interdisciplinary consultation

Establish clear triggers for physician-dentist communication, such as prior to initiating bisphosphonate therapy, before cardiac valve surgery, or when managing patients with poorly controlled diabetes (108).

#### 7.5.3 Integrated health record alert

In electronic health records, create an automated alert or reminder for patients with a new CVD diagnosis to undergo a comprehensive periodontal examination within the next 6 months (109).

### 7.6 Considerations for personalized risk assessment: the role of sex differences

Translating these general guidelines into personalized patient care requires a deeper understanding of individual risk modifiers, among which biological sex is emerging as a critical factor.

Cardiovascular diseases exhibit well-known sex differences in terms of age of onset, prevalence, and potentially pathophysiology, with men generally developing atherosclerosis and experiencing cardiovascular events earlier in life than women (1). Similarly, the composition and function of the human microbiome, both oral and gut, are increasingly recognized as being influenced by biological sex (110). Periodontitis itself shows a higher prevalence in males compared to females (111). These observations raise the question of whether the link between the oral microbiome and atherosclerosis also exhibits sex-specific patterns.

A key study providing direct evidence for sex differences in this relationship comes from the Northern Finland Birth Cohort. Researchers investigated the association between oral microbiome characteristics and carotid intima-media thickness (cIMT) in middle-aged participants. After carefully excluding individuals with major CVD-influencing factors, they found significant associations between oral microbiome diversity and higher cIMT, but only in the male sub-cohort (1). No such association was observed in the female sub-cohort. Furthermore, specific bacterial genera showed significant positive or inverse associations with cIMT exclusively in males. This study strongly suggests a potential sex-specific interaction between the oral microbiome and the early stages of atherosclerosis.

The underlying reasons for these observed sex differences are likely multifactorial. Sex hormones (estrogens and androgens) are known to exert profound effects on both the immune system and microbial communities, thereby modulating the link between oral dysbiosis and atherogenesis (112). For example, estrogen receptors can modulate inflammatory responses to oral pathogens, and evidence suggests that the protective effect of estrogen on endothelial nitric oxide synthase (eNOS) expression is diminished in postmenopausal women, potentially enhancing their vulnerability to the vascular effects of pathogens like P. gingivalis (113).

This mechanistic complexity is reflected in recent large-scale epidemiological data. A 2023 meta-analysis encompassing 26 studies and hundreds of thousands of participants investigated sex-specific risks (114). While it found no significant sex difference in the overall risk for cardiovascular disease associated with periodontitis, a crucial subgroup analysis revealed that the risk for coronary artery disease (CAD) specifically was higher in men than in women. This nuanced finding suggests that while the overall risk may be comparable, the specific cardiovascular pathologies driven by oral dysbiosis might follow different trajectories in males and females. Host genetics may also play a sex-specific role, with studies identifying differential genetic loci associated with oral microbiome features in a sex-stratified manner (115).

The clear implication of these findings is the critical need for sex-stratified analyses in future research investigating the oral microbiome and cardiovascular disease (110). Averaging effects across sexes may obscure important differences or lead to misleading conclusions. The mechanisms linking oral microbiota to atherosclerosis may genuinely diverge between males and females, necessitating sex-specific investigation to understand the pathways involved fully. From a clinical perspective, understanding these sex differences could have significant implications for personalized cardiovascular risk assessment. If the oral microbiome contributes differently to CVD risk in men and women, then risk prediction models incorporating oral biomarkers might need to be tailored by sex (116).

### 7.7 Models for integrated care and emerging therapeutics

The successful implementation of these guidelines requires a fundamental shift away from fragmented care towards new models that foster seamless collaboration. A real-world example is the collaboration between the Italian Society of Periodontology (SIdP) and the Italian Society of Hypertension, which outlines an integrated clinical approach emphasizing joint risk factor assessment and formal referral pathways (117, 118). While effective, the widespread adoption of such models faces significant structural barriers, including separate financing, insurance, and electronic health record systems for medical and dental care (43).

Looking forward, research is moving beyond traditional mechanical therapy to explore novel approaches that directly target the microbiome. A revolutionary frontier is the development of engineered microbial therapeutics (119, 120). For example, a groundbreaking preclinical study demonstrated that daily oral administration of an engineered *E. coli* Nissle 1917 strain, designed to continuously secrete short-chain fatty acids (SCFAs), significantly reduced myocardial injury in a mouse model of ischemic heart disease (121). This approach represents a shift towards using engineered probiotics as "living factories" to deliver cardioprotective molecules directly to the host.

The clinical translation of such strategies is becoming increasingly viable. Regulatory bodies like the U.S. FDA have established frameworks for Live Biotherapeutic Products (LBPs), and several microbiome-based therapeutics have already received approval for other conditions (e.g., Rebyota™, VOWST™) (122, 123). This paves the way for the next generation of precision microbiome interventions aimed at reducing cardiovascular risk, moving the field from association to targeted, mechanism-based prevention and therapy.

## 8 Conclusion

The relationship between the oral microbiome and atherosclerosis has evolved from a field defined by epidemiological association to one characterized by deep mechanistic inquiry. The evidence synthesized in this review underscores a paradigm shift, where the focus has moved from identifying specific pathogenic bacteria to understanding the functional consequences of a dysbiotic oral ecosystem. New research frontiers are providing unprecedented insight. Integrative multi-omics are revealing complex functional signatures that transcend simple taxonomic lists, while the study of epigenetics provides a compelling molecular basis for the long-term persistence of cardiovascular risk via "inflammatory memory" (118, 124). Furthermore, the oral-gut-vascular axis has emerged as a primary, unified pathway through which oral pathobionts can exert profound systemic effects (9).

However, this rapid scientific progress must be viewed through a lens of critical appraisal. The existing literature is hampered by significant methodological heterogeneity, which has led to inconsistent findings and complicates the synthesis of evidence (17). Moreover, a substantial translational gap exists due to fundamental differences between preclinical animal models and human physiology. Most critically, the question of causality remains elusive. The lack of definitive evidence from RCTs on hard clinical endpoints, combined with conflicting results from Mendelian randomization studies and the failure to satisfy formal causality criteria, indicates that a direct, linear causal relationship for major atherosclerotic events is not supported by the current highest levels of evidence.

Despite these challenges, the weight of the evidence has catalyzed a consensus among leading cardiology and periodontology societies, resulting in landmark joint clinical guidelines. These frameworks, and the integrated care models they inspire, are paving the way for a more holistic approach to patient management by breaking down the traditional silos between medicine and dentistry. The future of the field lies in addressing the current evidence gaps through large-scale, methodologically rigorous RCTs and advancing novel interventions, such as engineered microbial therapeutics, from preclinical promise to clinical reality. Harnessing our growing understanding of the oral-systemic connection is a critical step towards developing the next generation of personalized strategies for cardiovascular risk reduction.

## Glossary

Aa: *Aggregatibacter actinomycetemcomitans*
CAD: Coronary Artery Disease
CAP: Chronic Apical Periodontitis
CAS: Carotid Atherosclerosis
cIMT: Carotid Intima-Media Thickness
CRP: C-Reactive Protein
CVD: Cardiovascular Disease
DNMT1: DNA Methyltransferase 1
eNOS: Endothelial Nitric Oxide Synthase
Fn: *Fusobacterium nucleatum*
HAT: Histone Acetyltransferase
HSP: Heat Shock Protein
IL: Interleukin
LBP: Live Biotherapeutic Product
LPS: Lipopolysaccharide
MA: Meta-Analysis
MACE: Major Adverse Cardiovascular Events
MI: Myocardial Infarction
MMP: Matrix Metalloproteinase
MR: Mendelian Randomization
NSPT: Non-Surgical Periodontal Therapy
OR: Odds Ratio
PAD: Peripheral Artery Disease
PD: Periodontal Disease
Pg: *Porphyromonas gingivalis*
RCT: Randomized Controlled Trial
SCFA: Short-Chain Fatty Acid
SES: Socioeconomic Status
Spp.: Species (plural, used in taxonomy)
TLR: Toll-Like Receptor
TMAO: Trimethylamine N-oxide
TNF-α: Tumor Necrosis Factor-alpha
VSMC: Vascular Smooth Muscle Cell
WMD: Weighted Mean Difference
XAI: eXplainable Artificial Intelligence
